# Potential Neuroprotective Role of Melatonin in Sepsis-Associated Encephalopathy Due to Its Scavenging and Anti-Oxidative Properties

**DOI:** 10.3390/antiox12091786

**Published:** 2023-09-21

**Authors:** Mariusz Sieminski, Karolina Szaruta-Raflesz, Jacek Szypenbejl, Klaudia Krzyzaniak

**Affiliations:** Department of Emergency Medicine, Medical University of Gdansk, 80-214 Gdansk, Poland; karolina.szaruta@gmail.com (K.S.-R.); klaudiak@gumed.edu.pl (K.K.)

**Keywords:** melatonin, sepsis-associated encephalopathy, sepsis, antioxidant, neuroinflammation

## Abstract

Sepsis is defined as life-threatening organ dysfunction caused by a dysregulated host response to infection. The brain is one of the organs involved in sepsis, and sepsis-induced brain injury manifests as sepsis-associated encephalopathy (SAE). SAE may be present in up to 70% of septic patients. SAE has a very wide spectrum of clinical symptoms, ranging from mild behavioral changes through cognitive disorders to disorders of consciousness and coma. The presence of SAE increases mortality in the population of septic patients and may lead to chronic cognitive dysfunction in sepsis survivors. Therefore, therapeutic interventions with neuroprotective effects in sepsis are needed. Melatonin, a neurohormone responsible for the control of circadian rhythms, exerts many beneficial physiological effects. Its anti-inflammatory and antioxidant properties are well described. It is considered a potential therapeutic factor in sepsis, with positive results from studies on animal models and with encouraging results from the first human clinical trials. With its antioxidant and anti-inflammatory potential, it may also exert a neuroprotective effect in sepsis-associated encephalopathy. The review presents data on melatonin as a potential drug in SAE in the wider context of the pathophysiology of SAE and the specific actions of the pineal neurohormone.

## 1. Introduction—Sepsis and Sepsis-Associated Encephalopathy

### 1.1. Sepsis

Sepsis is a life-threatening complex of biochemical and clinical abnormalities caused by infection. The actual definition of sepsis was devised in 2016 and describes this syndrome as “life-threatening organ dysfunction caused by dysregulated host response to infection” [1]. The diagnostic workup should start with the clinical suspicion of infection (e.g., fever, symptoms of upper respiratory airway infection, dysuria) followed by screening with the Quick Sequential Organ Failure Assessment (qSOFA) scale. In cases of positive screening, a wider assessment of multi-organ failure should be performed with the Sequential Organ Failure Assessment (SOFA) scale. A score of ≥2 points in the SOFA is equivalent to a diagnosis of sepsis [1,2].

Sepsis is a severe problem for healthcare systems, with a global incidence of 189/100,000 person-years in the population of hospitalized adults and with mortality of 26.7% [3]. Rhee et al. found that sepsis was diagnosed in 6% of patients admitted to hospitals in the USA, with mortality of 15% and with 6% of patients requiring palliative care after discharge [4]. The survivors of sepsis frequently struggle with troublesome chronic consequences—in 74% of them, a new co-morbidity is diagnosed within the first year after hospital discharge, and one third of them become dependent on nursing care after surviving sepsis [5]. It is of special note that in 18.5% of sepsis survivors, a diagnosis of cognitive dysfunction was made. These data show the importance of any intervention that potentially might reduce mortality and post-septic disability and have a neuroprotective effect.

### 1.2. Sepsis-Associated Encephalopathy

Sepsis is defined as the failure of organs resulting from a pathological inflammatory reaction to infection. The central nervous system (CNS) is one of the most vulnerable organs due to its circulatory and metabolic needs. The systemic inflammatory process causes dysfunction of the blood–brain barrier, allowing the influx of proinflammatory mediators to the CNS, resulting in the spreading of inflammation across the cerebral structure. Sepsis-related hypotonia decreases cerebral perfusion, which leads to a deficiency of metabolic substrates for neural tissue. Therefore, symptoms of involvement of the CNS develop rapidly during sepsis, being the first clinical symptoms of sepsis in some cases. Sepsis-associated encephalopathy (SAE) is a term describing brain failure caused by sepsis in the absence of direct brain injury or infection of the central nervous system. It must be remembered that there is no formal clinical definition of SAE or formal diagnostic criteria. Biochemical, neurophysiological or radiological markers of SAE are lacking as well. Therefore, the diagnosis of SAE is made on clinical grounds exclusively. The diagnosis is based upon the clinical observation of cognitive deterioration temporarily related to symptoms of sepsis, with the exclusion of direct brain injury and neuroinfection.

SAE is a diffuse cerebral dysfunction [6]. The clinical spectrum of SAE ranges from discrete cognitive deficits, e.g., reduced attention or diminished readiness for social interactions (so called sickness behavior) [7] through delirium to coma [8]. Most commonly, acute SAE is observed, but its subacute (lasting for weeks) or chronic (lasting for months) forms may be observed. The exact pathophysiological mechanism of SAE remains unknown, with many potential pathways leading to this phenomenon. The most important pathways leading to the development of SAE are dysfunction of the BBB, neuroinflammation, oxidative stress and cerebral ischemia. The development of SAE is a consequence of the dysfunction of the brainstem, frontal cortex and amygdala, as well as of the failure of neuroendocrine centers: the hypothalamus and pituitary gland [9]. Dysfunction of the CNS is reflected by pathological patterns on an electroencephalogram (EEG), which is characterized by the presence of triphasic waves, theta waves or burst suppression [10].

### 1.3. Clinical Picture of SAE

Sepsis-associated encephalopathy may be categorized as a cognitive and communicative disorder, at least in its mild forms. Patients present problems with concentration, reduced attention and problems with learning and remembering new facts [11]. These cognitive deficits are accompanied by irritability, depressed mood, anxiety and withdrawal from social activities—a spectrum of symptoms defined as sickness behavior [7]. The clinical progression of SAE leads to the appearance of confusion and further features of delirium with disorders of consciousness. These symptoms may develop acutely or sub-acutely, with the patient being disoriented, agitated and aggressive, with hallucinations and a tendency to wander and speak loudly [6,12], as occurs in hyperactive delirium. Patients with SAE presenting as hypoactive delirium are less “visible”: they are quiet, apathetic and pathologically somnolent and show problems with the perception of environmental stimuli [12]. It is of note that this “silent” phenotype of delirium is more common in SAE, which makes detection of the disorder more challenging [13]. Although SAE is defined by a lack of focal brain lesions, the appearance of focal neurological deficits is described in approximately one fifth of patients and seizures may be present in 10% of subjects [14]. The progression of sepsis and development of septic shock with multi-organ failure increase the severity of SAE, finally leading to a coma.

Disturbed functioning of the central nervous system is reflected with a variety of changes in the EEG. Typical changes for septic encephalopathy are the domination of theta or delta rhythms, the presence of triphasic waves and burst suppression [15,16]. Epileptic discharges and pseudo-epileptic discharges are recorded in nearly 10–50% of patients with sepsis, with most seizures being non-convulsive [10,13,17].

The clinical diagnosis of SAE is paralleled with various findings in neuroimaging studies. Volumetric analyses revealed that SAE leads to brain atrophy, with a visible reduction in the volume of the white matter, cortex hippocampus and amygdala [18]. Disturbances of the cerebral blood flow have also been descried in septic patients [19]. Disseminated ischemic lesions are most often found in septic patients [14]. A noticeable percentage of septic patients develop radiological features of posterior reversible encephalopathy syndrome (PRES) [20].

### 1.4. Epidemiology of SAE

As was mentioned earlier, there are no strict, widely agreed and validated diagnostic criteria for SAE. One of the consequences of this fact is the lack of precise data on the incidence and prevalence of SAE in the population of septic patients. The results of epidemiological studies significantly differ according to the definition of SAE used. The most commonly cited figure of the 70% prevalence of SAE among septic patients comes from a study by Young et al. published in 1990. This prospective study included 69 septic patients and indeed the presence of encephalopathy was found in 70% of them [21]. It must be noted that this study was performed before contemporary definitions of sepsis were devised and therefore data from recent studies should be closer to the actual state. Chen et al., in their retrospective analysis performed in one center (defining SAE as the presence of cognitive and neuropsychiatric disorders in septic patients with no history or chronic neurological disorders or features of metabolic encephalopathy), found a prevalence of 43.6% of SAE. Patients with encephalopathy were older, with no significant differences in gender distribution, had higher scores on the APACHE II and SOFA scales and were at higher risk of in-hospital death [22]. These results are in concordance with those reported by Feng et al. These authors found an incidence of SAE of 42.3% septic patients, and subjects with SAE had higher scores of APACHE II and SOFA and higher mortality rates [23]. Sonneville et al. performed a retrospective multi-center study and found a slightly higher prevalence—53% of septic patients had SAE [24]. Therefore, it may be assumed that about half of all septic patients suffer from encephalopathy and that this condition is related to a more serious course of sepsis and higher mortality.

A frequently neglected fact is that acute sepsis-related brain injury may transform into a chronic form, becoming a cause of neurological and cognitive decline. A prospective study performed by Iwashyna et al. showed that in 10% of sepsis survivors, moderate and severe cognitive deficits were diagnosed de novo [25]. Another study showed that in a population of elderly subjects who survived 3 years after the diagnosis of sepsis, there were approximately 15% of patients with moderate and severe cognitive deficits [26]. In other studies, the prevalence of cognitive disorders reached over 20% [27]. Recently, it was also observed that in a middle-aged population, sepsis increases the rate of cognitive decline [28].

The high prevalence of sepsis-associated encephalopathy, its negative impact on prognosis and its chronic cognitive consequences (especially in the context of an increasing number of sepsis survivors) make disentangling the mechanisms of SAE and finding therapies with the potential to reduce the consequences of SAE urgently needed.

### 1.5. Melatonin

Melatonin is a neurohormone widely spread in nature, found both in plant and animal species. The most important source of melatonin in humans is the pineal gland, where this neurohormone is produced with the circadian rhythm, but multiple local sources of melatonin also exist, like the retina [29], immune cells [30], skin [31] or the epithelium of the gastrointestinal tract [32]. The most noticeable physiological role of melatonin in humans is the control of the circadian rhythm [33,34], but it exerts numerous other effects. Melatonin is a potent antioxidant [35] and anti-inflammatory factor [36]. It also has an impact on energy metabolism [37] and on the cardiovascular system [38].

The above-mentioned properties of melatonin make it an important factor influencing the inflammatory response. This is why so much attention has been given recently to the role of melatonin in sepsis. The aim of our review is to analyze the available literature on the potential neuroprotective effect of melatonin in sepsis-associated encephalopathy (SAE).

## 2. Mechanisms of Sepsis-Associated Encephalopathy

### 2.1. Pathomechanisms of SAE

The pathomechanism of SAE is not completely understood. It is certain that its etiology is multifactorial, with blood–brain barrier disruption, neuroinflammation, ischemia and oxidative stress playing crucial roles.

Sepsis-associated encephalopathy is diagnosed only in situations where the central nervous system is not infected. The systemic inflammatory response continues outside the brain, being hidden behind the blood–brain barrier (BBB). The BBB is a highly integrated “wall” built of endothelial cells, pericytes, astrocytes and microglial cells. Thanks to the BBB, most molecules may pass into the brain only through controlled transcellular transport—this also includes inflammatory mediators. The BBB also limits the migration of peripheral cells of the immune system to the CNS [39]. Therefore, an increase in the permeability of the BBB may be considered as the first step towards the involvement of the brain in sepsis and the development of SAE. Indeed, it has been found in a post-mortem study that proteins (occludin, claudin-5, ZO-1) forming so-called tight junctions between endothelial cells within the BBB were practically absent in the brains of septic patients. This means that during sepsis, the permeability of the BBB is significantly increased [40]. This allows the influx of proinflammatory cytokines into the central nervous system, which is followed by leukocyte infiltration—the systemic inflammatory response invades the brain. The next step is the activation of localized microglia within CNS immunocompetent cells [41]. It has been shown in humans that the activation of microglial cells is related to the development of delirium [42]. Microglial cells activated by proinflammatory cytokines entering the CNS through the pathologically permeable BBB present a proinflammatory phenotype [43] and start to produce their own proinflammatory cytokines—mainly tumor necrosis factor alpha (TNF alfa), interleukin-1 beta (Il-1b) and interleukin-6. The presence of these cytokines, along with the release of reactive oxygen species, nitric oxide and glutamate, further stimulates the neuroinflammatory process, closing a vicious circle of neuroinflammation, leading to the progressing damage of neural cells and to cell death through a mechanism called pyroptosis [44,45]. This injury of neural tissue leads to clinical symptoms of encephalopathy and delirium.

Parallel to the neuroinflammatory process, the septic brain struggles with acute disturbances within the supply chain of metabolic substrates. Sepsis and septic shock are characterized by perturbances in systemic circulation, hypotonia being the most prominent of them [46]. Simultaneously, it was shown that the autoregulation of cerebral arteries is less efficient in septic patients [47,48,49]. An active inflammatory process is also a pro-thrombotic condition. Regardless of the dysregulated blood flow, the cerebral arteries are blocked with clots in septic patients. Thus, another factor increasing the severity of sepsis-related brain injury is disseminated focal ischemia of the brain, leading to cellular death or significant metabolic perturbances, as the oxygenation of the brain tissue is significantly reduced in sepsis [50]. Therefore, the features of brain ischemia were present in all septic patients in one neuropathological study [51].

Neuroinflammation, BBB disruption and ischemia lead to the malfunction of neural cells, which in turn is the cause of pathological neurotransmission. The most widely studied is the cholinergic pathway—its anti-inflammatory action is suppressed in sepsis [52]. Sepsis-related deficiency in dopaminergic transmission is also suggested by some publications [53,54]. Proinflammatory cytokines (Il-1B) may increase the biological effect of GABA-ergic transmission, leading to pathological somnolence or cognitive disorders [55].

**Figure 1:**
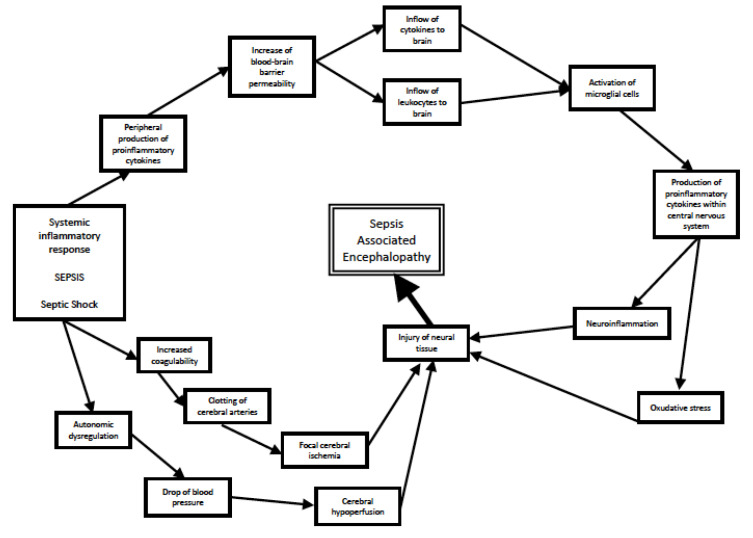
Processes leading to development of SAE.

Summing up, disruption of the BBB allows the influx of proinflammatory cytokines into the CNS, which leads to microglial cells’ activation and the initiation of a neuroinflammatory vicious cycle (activated cells produce more cytokines, which activate other cells, etc.). The neuroinflammation causes damage and death in neural cells, which is one of the biological substrates in the symptomatology of SAE. Further dysfunction of the brain is caused by sepsis-related hemodynamical instability and hypercoagulability, leading to ischemia. Neuroinflammation and ischemia disturb the normal balance of neurotransmitters, which is another cause of disorders of cognition and consciousness typical of SAE. The most important processes leading to SAE are presented in Figure 1.

### 2.2. The Role of Oxidative Stress and Free Radicals in Sepsis-Associated Encephalopathy

All the above-mentioned processes constitute a serious metabolic challenge to the cells of the central nervous system. The mitochondria of neurons must work in circumstances of increased energetic needs resulting from the high activity of the inflammatory process and of a decreased supply of oxygen and glucose, resulting from ischemia of the CNS. This leads to oxidative stress and mitochondrial dysfunction [56]. In vivo models of lipopolysaccharide-evoked sepsis-associated encephalopathy show that neuroinflammation leads to mitochondrial damage in endothelial cells through the pathological activation of dynamic-related protein 1 (Drp1), which was correlated with increasing mitochondrial oxidative stress and the loss of mitochondrial membrane potential. These phenomena were correlated with the reduced expression of proteins responsible for the formation of tight junctions between endothelial cells (ZO-1 and occludin) and thus leading to the increased permeability of the BBB. Mitochondrial damage was also detected in neuronal cells, with a decrease in oxidative phosphorylation, increased glycolysis and a reduction in the mitochondrial membrane potential and production of ATP. The final consequence of this process is the death of neurons [57]. Other animal studies have also shown the increased production of reactive oxygen species (ROS), reduced production of ATP and intense apoptosis [58]. Activated microglial cells also generate reactive oxygen species (ROS) and reactive nitrogen species (RNS), which finally leads to neuronal injury and death [59]. Oxidative stress, mitochondrial dysfunction and the presence of ROS and RNS trigger proapoptotic cellular caspase-dependent pathways, leading to the death of cells, which is the histological substrate for the clinical symptoms of SAE and its chronic consequences [60]. This is why any therapeutical intervention with the potential to reduce oxidative stress and scavenge reactive species may be neuroprotective and limit the chronic cognitive deficits in survivors of sepsis. This was already shown in animal models with anti-oxidative therapies, e.g., with molecular hydrogen [61] or tetramethylpyrazine [58].

## 3. Clinical Role of Melatonin

Melatonin is frequently mentioned as a potential drug in septic patients. The antioxidant, anti-inflammatory and neuroprotective properties of this hormone, which are described in detail below, make it a very interesting potential therapeutic factor in patients with sepsis and SAE.

### 3.1. Melatonin

Melatonin is a neurohormone synthesized mainly in the pineal gland. It is a tryptophan derivative, N-acetyl-5-methoxytryptamine. Apart from the pineal gland, it is also produced in the retina, skin, gastrointestinal tract and in cells of the immunological system [62]. It is produced and released in the circadian rhythm, with the lowest concentrations during the day (<2 pg/mL) and highest during the night (approximately 100 pg/mL) [63]. It has been also discovered that in certain conditions, the local tissue concentration of melatonin may be significantly higher than in plasma [64]. It acts through two G-protein-coupled receptors, MT1 and MT2, most abundantly met in the pars tubercles of the pineal gland, retina and hypothalamus [65]. Nevertheless, it must be noted that most of the anti-inflammatory and antioxidant actions of melatonin are independent of MT1 and MT2 and are exerted through the activation of NRF2-related pathways, as was shown by Janjetovic et al. [66].

### 3.2. Main Clinical Roles of Melatonin

The main physiological role of melatonin is the regulation of the circadian rhythm. In the case of humans, this means promoting sleep during the dark part of the day. The production and secretion of melatonin is inhibited by light through the stimulation of the retina and activation of the suprachiasmatic nucleus [67]. Its production is increased in darkness, when melatonin may fully exert its physiological role, which is switching the organism to “sleep mode” [68]. The localization of melatonin receptors suggests that the neurohormone acts through the modulation of the function of neural and neuroendocrine centers but also through direct impacts on the systems and organs of the body [62,67]. The chronobiological role of melatonin is expressed through driving the rhythm of the functioning of peripheral tissues and organs, adjusting them to the day–night cycle [69]. This role is crucial in terms of sepsis and the development of SAE. Sleep/wake rhythm disorders are frequently reported in septic patients and their presence negatively impacts prognosis [70]. On the other hand, a strong and probably causal relation between disrupted circadian rhythms and the development of delirium/encephalopathy in critically ill patients has been observed [71]. A low concentration of melatonin was discovered in septic patients with disordered circadian rhythms, which suggests a potential therapeutical role of this neurohormone at least as a circadian rhythm keeper [72]. It must be noted that the usage of melatonin (as a hypnotic drug) in intensive care units has increased in some countries [73]. As “switching to sleep mode” requires the modulation of the activity of the autonomic nervous system, it was proven that melatonin has potential to reduce the sympathetic drive [74]. This potential to modulate the activity of the autonomic nervous system was elegantly shown in patients who underwent pinealectomy—a therapy with exogenic melatonin restored the sympathetic–parasympathetic balance in these subjects [75]. This ability to harmonize both parts of the autonomic nervous system may be especially important in septic patients, in which the disruption of sympathovagal modulation may even precede the development of a systemic inflammatory response [76]. It can be speculated that the use of melatonin may be parallel to the stimulation of the vagal nerve, as successfully implemented in animal models of sepsis [77]. The impact of melatonin on the autonomic nervous system manifests also through the influence of melatonin on the values of blood pressure. Melatonin reduces vasoconstriction and blood pressure, and it is proposed as an adjunct drug in the treatment of hypertension [78]. Melatonin has also a significant impact on metabolism, being an important protective factor against obesity and the development of diabetes mellitus [37,79,80]. Melatonin also plays a role in modulating the inflammatory response, having noticeable anti-inflammatory potential, as it can prevent cell death caused by pyroptosis [81,82]. The above-listed functions of melatonin (autonomic and blood pressure control, metabolism control, modulation of inflammatory response) make it a very important neurohormone and potentially a therapeutic agent in septic patients.

### 3.3. Scavenging and Antioxidant Potential of Melatonin

As mentioned above, sepsis and SAE are related to the increased production and activity of free radicals. Neuroinflammation, being the most important putative factor of SAE, leads to the very intense production of oxidants within the central nervous system, with normal or decreased abilities for their consumption. This situation fulfills the definition criteria for oxidative stress [83]. Oxidative stress and high concentrations and activity of free radicals lead to the damage of vital molecules such as DNA, phospholipids and proteins, which in turn exert pro-apoptotic action and cause damage to biological membranes, with the fragmentation and death of cells. Cells and tissues can be protected from oxidative stress by molecules called antioxidants (molecules promoting processes that diminish the production of free radicals and intensify the consumption of oxidants) and scavengers (molecules capable of trapping free radicals directly) [62].

Melatonin has significant antioxidant potential. First, it can scavenge some of the free radicals directly. It has been shown that melatonin can react with hydroxyl (OH) radicals [84,85]. Melatonin may also serve as a substrate of reaction, leading to the elimination of peroxyl radicals [86,87] as well as nitric oxide [88].

Apart from the direct scavenging of free radicals, melatonin is capable of suppressing their production through the chelation of metal ions (e.g., Cu, Fe) necessary for the synthesis of oxidants. It was proven that melatonin chelates metal ions—melatonin was able to create complexes with copper, iron, cadmium or aluminum, blocking the participation of these ions in oxidant-producing reactions [89]. Therefore, melatonin is able to protect tissues from oxidative, metal-catalyzed damage [90,91].

Moreover, melatonin may participate in the repair of oxidative molecular damage. Colares et al. have recently proven that treatment with melatonin reduces the damage of DNA in cirrhotic rats [92]. Therapy with melatonin also intensifies the repair of DNA in neoplasmatic cells [83]. Melatonin was shown to reduce oxidative DNA damage in neural tissue caused by ischemia or trauma [93,94].

Lastly, melatonin activates antioxidant enzymes and influences signaling pathways involved in the generation of free radicals. Gou et al. described the protective effect of melatonin on neural tissue in hypoxic–ischemic brain damage exerted through the modulation of pathways leading to the activation of glutathione peroxidase—an antioxidant enzyme [95]. The melatonin-driven activation of antioxidant enzymes is a well-established fact [96].

It is also noteworthy that the metabolites of melatonin are potent antioxidants that increase the therapeutical potential of the pineal neurohormone. These metabolites may counteract environmental stresses, including oxidative stress, as was described in a review by Slominski et al. [97]. The main metabolites of melatonin, like N^1^-acetyl-N^2^-formyl-5-methoxykynuramine (AFMK), N-acethyl-5-methoxykynuramine (AMK), 6-hydroxymelatonin and 4-hydroxymelatonin, act as direct scavengers, metal-chelating agents or molecules capable of repairing oxidative damage, as was presented in a review by Galano and Reiter [98].

As this short summary shows, both endo- and exogenic melatonin may serve as a potent antioxidant protectant, reducing the oxidative tissue damage evoked by sepsis and neuroinflammation.

## 4. Role of Melatonin in Sepsis and in Sepsis-Associated Encephalopathy

### 4.1. Therapeutic Potential of Melatonin in Sepsis

Sepsis is a dysregulated response to infection, resulting from the overproduction of proinflammatory cytokines, extreme oxidative stress and the progressing failure of the organs and systems of the organism. As was shown in numerous studies, melatonin has the potential to exert a multidirectional therapeutical impact, protecting the tissues against the consequences of sepsis.

As was discussed above, melatonin is a powerful antioxidant. Zhen et al., in a recent study, found that melatonin could increase the activity of the antioxidant enzyme superoxide dismutase in the myocardium of septic rats, which led to less oxidative tissue damage [99]. Rahim et al. published similar results based upon an animal model of sepsis, showing that melatonin increases antioxidative defense through NRF2 activation [100]. Melatonin is able to activate NRF2, which in turn increases the expression of antioxidative enzymes (like SOD), which may prevent multiorgan failure in sepsis. Kang et al. proved that melatonin may prevent the development of lung injury through this pathway [101]. The melatonin-induced activation of the NRF2 signaling pathway was also shown to exert neuroprotective action in a model of the lipopolysaccharide-induced injury of neural tissue (analogous to models of septic encephalopathy) [102]. Melatonin is also capable of upregulating the activity of sirtuins (especially SIRT1 and SIRT 3) and thus leads to increased antioxidant activity of superoxide dismutase [103]. In this specific experiment, melatonin was shown to protect the small intestine from sepsis-evoked oxidative injury.

One typical pathological phenomenon in sepsis is mitochondrial dysfunction leading to energetic failure [104]. It was shown that melatonin may reverse sepsis-related pathological changes within mitochondria. The infusion of melatonin was shown to improve mitochondrial respiration and to reduce oxidative stress. This effect was paralleled with the reduction of organ failure markers [105]. Escames et al. found that melatonin exerted a bidirectional therapeutical effect upon mitochondria in septic mice: the pineal neurohormone inhibited mitochondrial nitric oxide synthase, reducing oxidative stress and simultaneously restoring the production of ATP [106].

Melatonin influences also the interplay between pro- and anti-inflammatory cytokines. Carillo-Vico et al. found that in a rat model of septic shock, melatonin decreased the concentration of the proinflammatory IL-10 and TNF-alpha, increasing simultaneously the concentration of anti-inflammatory IL-12 [107]. Melatonin may also be a crucial player in modulating the inflammatory response through the inhibition of the action of the inflammasome NLRP3 [108].

Promising results of studies performed on animal models and observations of a relation between low concentrations of melatonin and sepsis-related mortality [109] led to the first human trials. Alamili and colleagues induced endotoxemia in healthy human volunteers who were pre-treated with melatonin or a placebo. The researchers observed that melatonin (compared with placebo) led to a reduction in the concentration of some proinflammatory cytokines (e.g., interleukin Il-1beta) during the day, with no statistically significant effect during the night [110,111]. Galley et al. performed a study with a human whole blood model of sepsis, proving that melatonin reduced oxidative stress and mitochondrial failure as well as the production of proinflammatory cytokines [112]. Aisa-Alvares and co-workers performed a small, single-center clinical trial in septic patients, assessing the impact of various antioxidant molecules. Melatonin appeared to reduce the concentration of procalcitonin and lipid peroxidation [113]. Melatonin was also shown to reduce the need for mechanical ventilation and for the use of a vasopressor in another small (*n* = 40 patients), single-center clinical trial [114]. Mansilla-Roselló et al. published very recently the results of a clinical study comparing the 5-day therapy of septic patients with the i.v. infusion of 50 mg of melatonin versus a placebo. The authors observed that patients treated with melatonin had significantly shorter hospital stays, lower values of the SOFA score, lower concentrations of procalcitonin and more intense antioxidant activity [115]. The clinical usefulness of the antioxidant properties of melatonin was also shown in a recent clinical study focusing on septic patients, which compared the effectiveness of 50 mg of orally given melatonin with other antioxidants. It was shown that melatonin reduced the SOFA score and lipid peroxidation while increasing the total antioxidant capacity [116]. Details of all the above-mentioned clinical studies are presented in Table 1. Although the results are very promising, the limitations of the studies must be noted. All of them were single-center studies with a limited number of patients included. This does not allow the generalization of the conclusions. Multi-center studies with the randomization of large groups of patients are required.

Human studies on melatonin in sepsis were performed with a variety of dosages, ranging from 50 mg p.o. daily to 100 mg i.v. daily. Establishing the proper dosage of a neurohormone that is released in a specific circadian rhythm is challenging task. Galley et al. undertook this and performed a clinical study aiming at assessing the clinical safety, tolerance and optimal dosing of melatonin. There were 10 patients with sepsis resulting from community-acquired pneumonia participating in the study. Five of them received 50 mg of melatonin p.o. daily and the other five were given 20 mg of the neurohormone daily. Clinical observation led to the conclusion that therapy with melatonin did not lead to any adverse event and was well tolerated. Twenty mg daily was found to be the optimal dose from a pharmacological point of view [117].

The implementation of melatonin in clinical practice must be performed cautiously as little is known about its potential interactions with other drugs, especially the ones used in critically ill subjects (e.g., sedatives, vasopressors, antibiotics or steroids). It is a consequence of the sporadic use of melatonin in clinical practice. On the other hand, the authors of clinical trials assessing melatonin in sepsis did not report any significant interactions with routine therapy.

### 4.2. Neuroprotective Potential of Melatonin in Sepsis-Associated Encephalopathy

Ji et al. performed an interesting experiment with the immediate or delayed treatment with melatonin for sepsis-related brain injury in a mouse model of sepsis. It was shown that while immediate therapy with melatonin led to an increase in survival with no positive neurobehavioral effect, the delayed therapy with melatonin caused a neurobehavioral improvement [118]. The potential (although speculative) explanation for this finding may be that melatonin given immediately protects the integrity of the BBB and thus prevents the development of further neuroinflammatory processes. Due to this, sepsis-related brain injury is diminished and this decreases mortality with no visible neurobehavioral effect. Meanwhile, melatonin given later may only have a neuroprotective effect in the already inflamed brain and this explains the neurobehavioral benefit. This observation is very important in a human and clinical context as it suggests that therapy with melatonin may be somewhat beneficial regardless of the moment at which it is started. The beneficial and diverse neurologic effects of therapy with melatonin are not surprising in the context of the multidirectional action of this neurohormone in sepsis. This multipotency of melatonin is also visible in terms of its neuroprotective effect in sepsis-associated encephalopathy. As was shown earlier in the paper, the first event leading to SAE is blood–brain barrier (BBB) leakage. Wang et al. have shown that pre-treatment with melatonin may sustain BBB integrity in lipopolysaccharide (LPS)-evoked sepsis in mice. The main reason for BBB damage in sepsis is the degradation of tight junction proteins (e.g., occluding or claudin-5), which was clearly stopped with melatonin in Wang and colleagues’ experiment. This effect was achieved through the antioxidant action of melatonin, which inhibited the expression of enzymes involved in the production of free radicals (specifically, the gp91 phox subunit of NADPH oxidase) [119]. The same group of authors suggested that the supplementation of melatonin may protect BBB integrity in older patients suffering from sepsis [120].

As was mentioned earlier, melatonin exerts an anti-inflammatory effect, which also includes the suppression of neuroinflammation. Zhou et al. analyzed the effects of therapy with melatonin on neuroinflammation in an animal model of sepsis (lipopolysaccharide-injected rats). The authors reported a significant reduction in the production of proinflammatory mediators by microglial cells and the upregulation of the expression of anti-inflammatory factors in melatonin-treated animals. Moreover, a prominent shift towards the anti-inflammatory phenotype M2 of microglial cells was observed. These changes resulted in reduced white matter damage and demyelination in treated animals [121]. Melatonin reduces the LPS-evoked increase in the production of proinflammatory cytokines in the brain (e.g., IL-6, TNF-alpha) and simultaneously normalizes the expression and activity of redox signaling molecules (e.g., SOD2) [122]. Interestingly, the simultaneity of the anti-inflammatory and antioxidant action of melatonin in models of sepsis-associated encephalopathy is repeatedly described. Zhao et al. observed that in septic animals, therapy with melatonin led to a reduction in cerebral concentrations of proinflammatory cytokines (e.g., TNF-alpha, IN-1beta) and at the same time to a reduction in oxidative stress (confirmed by observations of the activation of SOD and diminished production of malondialdehyde) [123].

Apart from blood–brain barrier damage, neuroinflammation and oxidative stress, the septic brain is also injured due to multifocal ischemia resulting from decreased cerebral perfusion and inflammation-evoked thrombotic events. According to the results of a recently published, placebo-controlled, double-blind clinical trial, therapy with melatonin may intensify the neurologic improvement in patients after ischemic stroke [124]. This finding is not surprising as the neuroprotective action of melatonin in ischemic brain injury was proven in animal models. This neuroprotective effect is exerted through intensive antioxidant action (e.g., through an increase in the expression of glutathione peroxidase or through the attenuation of endoplasmic reticulum stress) [95,125] and the suppression of inflammatory processes (e.g., through promoting the anti-inflammatory phenotype of microglia and through a reduction in the expression of proinflammatory cytokines) [126,127]. It was also observed that in models of ischemic brain injury, melatonin could improve the integrity of the blood–brain barrier, similarly to what was observed in sepsis [128].

The result of the mechanisms described above is the suppression of apoptotic processes and reduction in brain injury during sepsis, which suggests potential for melatonin-driven better neurological outcomes of sepsis-associated encephalopathy.

**Figure 2:**
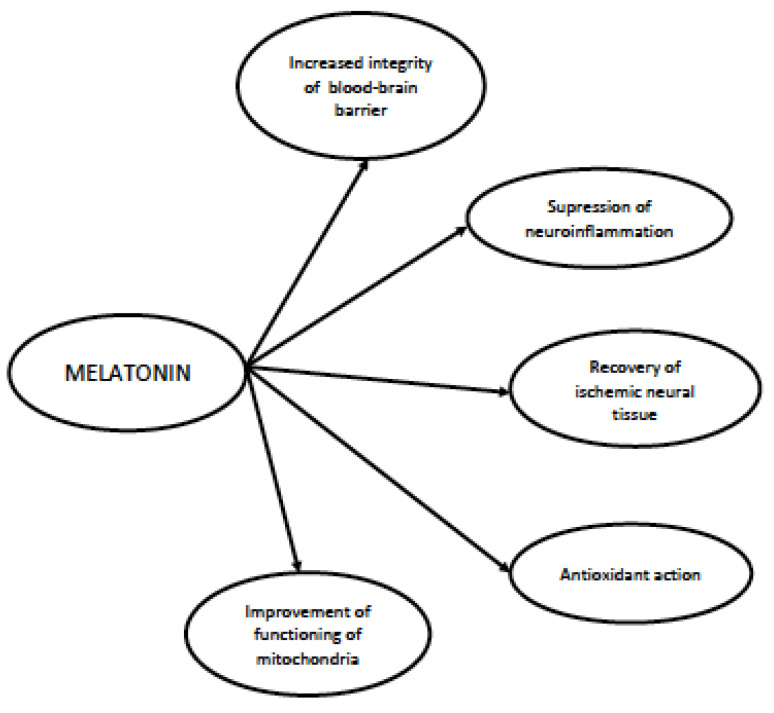
Potential therapeutical mechanisms of melatonin in sepsis and sepsis-associated encephalopathy.

The facts collected in the previous paragraphs suggest that melatonin may be a potent therapeutical agent in patient in sepsis and with SAE. This is strongly supported by the results of pre-clinical studies on animal models, which have found initial clinical confirmation in the human studies performed so far. The collected data allow us to plan more efficient protocols for clinical trials with melatonin in the upcoming future. Potential mechanisms through which melatonin may exert its effect in SAE are shown in Figure 2.

## 5. Conclusions and Future Perspectives

Melatonin is a fascinating neurohormone with a surprisingly wide range of potential actions. Some of them, like antioxidant potential, anti-inflammatory properties, a beneficiary impact on blood–brain barrier integrity or an ability to restore mitochondrial equilibrium, make this molecule a natural ally in the fight against sepsis and its complications and consequences. It must be remembered that the brain is the most vulnerable organ to circulatory, inflammatory and metabolic disorders caused by uncontrolled inflammation during sepsis. Sepsis-associated encephalopathy, being a consequence of blood–brain barrier leakage, neuroinflammatory processes, the injury of neural tissue caused by free radicals and multifocal ischemia of the brain, increases the mortality of septic patients and leads to chronic cognitive decline in a significant proportion of sepsis survivors. There is a significant group of molecules that may be potentially neuroprotective in SAE [129]. The data collected in this review suggest that melatonin may potentially act as a preventive or therapeutic substance in septic patients. On the other hand, we must be aware that regardless of the large amount of data coming from animal studies, clinical information on the therapeutic effectiveness of melatonin in humans is scarce. This constitutes the most important challenge for the future—implementing actual knowledge of melatonin in clinical trials in sepsis.

## Figures and Tables

**Table 1:** Clinical trials assessing usage of melatonin in sepsis.

Author of the Study (Year of Publication)	Number of Participants	Study Design, Dose of Melatonin Assessed	Assessed Outcome	Conclusions
Alamili et al. (2014) [110]	12 healthy volunteers with experimentally evoked endotoxaemia	Randomized, placebo-controlled, double-blinded cross-over trial, comparing infusion of 100 mg i.v. melatonin with placebo	Concentrations of pro- and anti-inflammatory markers in plasma of participants during night	Melatonin had no effect on concentratons of markers during the night
Alamili et al. (2014) [111]	12 healthy volunteers with experimentally evoked endotoxaemia	Randomized, placebo-controlled, double-blinded cross-over trial, comparing infusion of 100 mg i.v. melatonin with placebo	Concentrations of pro- and anti-inflammatory markers in plasma of participants during day	Melatonin reduced concentrations of pro-inflammatory biomarkers during the day
Aisa-Álvarez et. al. (2020) [113]	97 subjects divided into 5 subgroups	Randomized, controlled, triple-masked, and with parallel assignment clinical trial with a control group without treatment. Comparison of 4 molecules (vitamin C, vitamin E, n-acetylcysteine, melatonin—50 mg p.o. daily) and no antioxidant treatment	Concentrations of inflammation and oxidative stress markers in plasma	Reduction in procalcitonine and lipid peroxidation in subjects treated with melatonin, paralleled with reduction in sepsis severity measured with SOFA score
Taher et al. (2022) [114]	40 patients with septic shock	Prospective, two-arm, double-blind, randomized clinical trial; 50 mg melatonin given p.o. through 5 days was compared with placebo.	Change in SOFA score, need for mechanical ventilation, required dosage of vasopressor	Insignificant reduction in SOFA score, percentage of patients requiring ventilation and usage of vasopressors in melatonin-treated group
Mansilla-Roseló et al. (2023) [115]	29 patients (14 in placebo group, 15 in melatonin group)	Unicenter, randomized, placebo-controlled, double-blind trial; 60 mg of i.v. given melatonin was compared to placebo therapy	Change in SOFA score, concentrations of inflammatory markers, oxidative stress status	Treatment with melatonin led to decrease in SOFA score and in concentrations of inflammatory markers and improvement in oxidative stress status
Aisa-Álvarez et al. (2023) [116]	131 subjects divided into 5 subgroups	Randomized, controlled, triple-masked, and with parallel assignment clinical trial with a control group without treatment. Comparison of 4 molecules (vitamin C, vitamin E, n-acetylcysteine, melatonin—50 mg p.o. daily) and no antioxidant treatment	Change in SOFA score, change in concentrations of inflammatory markers and antioxidant activity	Therapy with melatonin was related to decrease in SOFA score, decrease in inflammatory markers and improvement in antioxidant activity

## Data Availability

Not applicable.
